# Antifungal Constituents of *Piper crocatum* and Their Activities as Ergosterol Biosynthesis Inhibitors Discovered via In Silico Study Using ADMET and Drug-Likeness Analysis

**DOI:** 10.3390/molecules28237705

**Published:** 2023-11-22

**Authors:** Tessa Siswina, Mia Miranti Rustama, Dadan Sumiarsa, Eti Apriyanti, Hirofumi Dohi, Dikdik Kurnia

**Affiliations:** 1 Department of Chemistry, Faculty of Mathematics and Natural Sciences, Universitas Padjadjaran, Sumedang 45363, Indonesia; tessa20001@mail.unpad.ac.id (T.S.); dadan.sumiarsa@unpad.ac.id (D.S.); eti.apriyanti@unpad.ac.id (E.A.); 2 Department of Midwifery, Poltekkes Kemenkes Pontianak, Pontianak 78124, Indonesia; 3 Department of Biology, Faculty of Mathematics and Natural Sciences, Universitas Padjadjaran, Sumedang 45363, Indonesia; mia.miranti.rustama@unpad.ac.id; 4 Graduate School of Horticulture, Chiba University, 1-33 Yayoi, Inage-ku, Chiba 263-8522, Japan; hdohi@faculty.chiba-u.jp

**Keywords:** *Piper crocatum*, antifungal, ergosterol, ADMET, drug-likeness analysis

## Abstract

Along with the increasing resistance of *Candida* spp. to some antibiotics, it is necessary to find new antifungal drugs, one of which is from the medicinal plant Red Betel (*Piper crocatum*). The purpose of this research is to isolate antifungal constituents from *P. crocatum* and evaluate their activities as ergosterol biosynthesis inhibitors via an in silico study of ADMET and drug-likeness analysis. Two new active compounds **1** and **2** and a known compound **3** were isolated, and their structures were determined using spectroscopic methods, while their bioactivities were evaluated via in vitro and in silico studies, respectively. Antifungal compound **3** was the most active compared to **1** and **2** with zone inhibition values of 14.5, 11.9, and 13.0 mm, respectively, at a concentration of 10% *w*/*v*, together with MIC/MFC at 0.31/1.2% *w*/*v*. Further in silico study demonstrated that compound **3** had a stronger ΔG than the positive control and compounds **1** and **2** with −11.14, −12.78, −12.00, and −6.89 Kcal/mol against ERG1, ERG2, ERG11, and ERG24, respectively, and also that **3** had the best Ki with 6.8 × 10^−3^, 4 × 10^−4^, 1.6 × 10^−3^, and 8.88 μM. On the other hand, an ADMET analysis of **1**–**3** met five parameters, while **1** had one violation of Ro5. Based on the research data, the promising antifungal constituents of *P. crocatum* allow *P. crocatum* to be proposed as a new antifungal candidate to treat and cure infections due to *C. albicans*.

## 1. Introduction

*Candida* spp. is part of the normal flora in healthy individuals and is usually confined to the skin and mucosal surfaces of the oral cavity, gastrointestinal tract, urogenital tract, and vagina; however, *Candida* spp. can cause a wide variety of infections on the mucosal surface under certain conditions [1,2]. Ergosterol, a cytochrome P450 enzyme in fungi descended from *S. cerevisiae* and a member of the CYP51 family, is a crucial enzyme in fungal-specific sterols and a primary target for antifungals because of its function in maintaining the function and integrity of *Candida* cell membranes [3,4]. The blocking of ergosterol biosynthesis as a result of lanosterol 14α demethylase (ERG11) inhibition will accumulate intermediate toxic sterol (14-methyl-3,6-diol) by ERG3 and cause impaired cell membrane, damage, and lysis [5,6,7].

Azole, polyene, echinocandin, and flucytosine (5-FC) are antifungal medications used today against *Candida* spp., while azoles target lanosterol 14α demethylase (CYP51), where the five-membered azole ring inhibits the biosynthesis of ergosterol and creates a coordination bond with iron heme, polyene works by forming complexes with the plasma membrane’s ergosterol, echinocandin inhibits β-glucan, and flucytosine prevents DNA synthesis, which prevents DNA replication from occurring [8]. Azoles are used because they have a broad spectrum of antimicrobials, but the fungal development of resistant strains and long-term use of antibiotics has caused azole antibiotic resistance, which has reached 83% in the Asia Pacific region and become a serious issue in recent years [9,10,11]. Based on this problem, the discovery of new antifungal agents from natural product resources is very important for solving the treatment and cure of fungal infections due to *C. albicans* [12].

One of the potential natural sources for a new antifungal agent is Red Betel leaf (*Piper crocatum* Ruiz and Pav), a herbal medicine that has traditionally been utilized for antifungal treatment and contains secondary metabolites of alkaloids, glycosides, saponin, tannin, phenol, triterpenoids, steroids, flavonoids, and essential oil [9,10], and the ethnopharmacology of *P. crocatum* data has shown its activities to be anti-microbial, antifungal, anti-oxidant, anti-bacterial, anti-inflammatory, analgesic, immunomodulatory, anti-tumor, anti-allergic, anti-diabetic, and hypotensive, respectively [11,13]. This research reported the identification of potential antifungal agents against *C. albicans* from *P. crocatum* via isolation, separation, and purification with various chromatography techniques, continuing with the structural determination of active compounds using spectroscopic methods, then followed by a bioactivity study using in vitro disc diffusion, Minimum Inhibitory Concentration (MIC), and Minimum Fungicidal Concentration (MFC) together with in silico studies of molecular docking, ADMET, and drug-likeness analysis against squalene epoxidase (ERG1), sterol 8Δ isomerase (ERG2), lanosterol 14α demethylase (ERG11), and sterol 14Δ reductase (ERG24) as enzymes that play important roles in ergosterol biosynthesis.

## 2. Results

### 2.1. Structure Elucidation of Compounds ***1***–***3***

#### 2.1.1. Structure Determinations of Compound **1**

**Figure 1:**
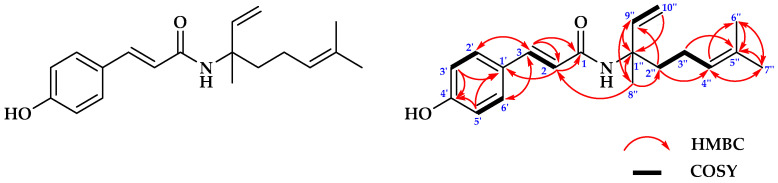
Structure of compound **1**, the HMBC, and ^1^H-^1^H COSY correlations.

Compound **1** was isolated as a colorless oil. The ^13^C-NMR and DEPT 135° spectra of **1** showed eighteen signals presented for three methyls at δc 17.7, 23.8, and 25.8 ppm; three methylenes at δc 22.5, 40.1, and 113.2 ppm; eight olefinic methines at δc 115.9, 115.9, 117.0, 123.9, 129.9, 129.9, 141.9, and 143.9 ppm; one quaternary carbon at δc 83.1 ppm; and four olefinic quaternary carbons at δc 127.4, 131.9, 157.7, and 166.5 ppm, respectively. The ^1^H–NMR spectrum showed three methyl proton signals at δ_H_ 1.60 (3H, s), 1.60 (3H, s), and 1.66 ppm (3H, s). The other proton signals identify as methylenes at δ_H_ 2.02 ppm (2H, dd, J = 7.5 and 16.25 Hz) and δ_H_ 1.83 and 1.91 ppm (2H, m) together with olefinic methylene at δ_H_ 5.16 ppm (2H, dd, J = 16.5 and 11 Hz), respectively. Further analysis indicated signals for ten methynes at δ_H_ 6.83 (1H, d, J = 8.5 Hz), 6.83 (1H, d, J = 8.5 Hz), 6.25 (1H, d, J = 16 Hz), 5.10 (1H, t, J = 2 Hz), 7.39 (1H, d, J = 8.5 Hz), 7.39 (1H, d, J = 8.5 Hz), 6.02 (1H, dd, J = 11 and 17.5 Hz), 7.54 (1H, d, J = 16 Hz), 1.71 (1H, s), and 5.87 ppm (1H, s), respectively. The four olefinic proton signals for an aromatic skeleton at δ_H_ 7.39 (2′ and 6′) and δ_H_ 6.83 ppm (3′ and 5′) with a coupling constant of J = 8.5 Hz indicated the orto positions of the benzene skeleton so that two substituents on the benzene were in the para position. Two other olefinic proton signals at δ_H_ 7.54 (3) and δ_H_ 6.25 ppm (2) with J = 16 Hz had a trans configuration that was identified bound to C2′ and C6′ (δc 129.9) and also to amide carbonyl C1 (δc 166.5), respectively, to form a framework of benzamine derivative. The detailed analysis showed that two methylene carbons at C2″-C3″, one olefinic carbon C4″, and one olefinic quaternary C5″ together with two methyl groups of C6″ and C7″ formed a long chain iso-propenyl framework. Supporting data derived from the ^1^H-^1^H-COSY spectrum showed the correlation between H-2 and H-3, H-2′ and H-3′, H-5′ and H-6′, H-9″ and H-10″ then H-3″ and H-2″ and H-4″, respectively. Further connectivity analysis of H and C to quaternary carbon C1″ at δc 83.1 ppm as an important carbon position for HMBC indicated that the connectivity of four sub-structures of prenyl, amides, benzene, and hydroxyl groups formed a complete suggested main framework structure, and the important HMBC correlated signals were observed for H-2″ to C4″ and C9″; H-3″ to C5″; H-4″ to C6″ and C7″; H-6″ to C5″ and C7″; H-7″ to C4″, C5″, and C6″; H-8″ to C1″, C2″, and C9″; H-9″ to C1″ and C8″; H-10″ to C1″; H-2 to C1 and C1′; H-3 to C1, C2, C2′ and C6′; H-2′ to C3; H-3′ to C1′ and C4′; and H-5′ to C1′ and C4′; while the H-6′ correlated to C3, respectively (Figure 1). According to the 1D and 2D-NMR data, compound **1** is suggested as an amide derivative attached to a hydroxyl group at C4′ (Table 1).

The presence of the functional group in **1** was identified from absorption signals of the infra-red (IR) spectrum at 3358, 2974 and 1169; 2930, 1374, and 756; 3358, 1683, and 1632; 1605, 1587, and 517; 1605, 1514, and 922; and 832 cm^−1^ for those corresponding to hydroxyl, methyl, carbonyl-amide, olefinic carbon, and the benzene ring in aromatic and alkene, sequentially, and also supported with the UV–Vis spectrum in those that showed three absorptions at 312, 227, and 211 nm and those correlated to the presence of O=C-NH, C=C=O and -OH, respectively. Finally, to confirm the proposed and final suggested structure of compound **1** derived from spectroscopic data of NMR, IR, and UV–Vis, its molecular weight was determined by mass measurement showed molecular weight (*m*/*z*) of compound **1** was 299.2908 (calcd. 299.2177 for C_19_H_25_NO_2_) based on the HR-TOFMS [M+H]^+^ ion peak in mass spectrometry ions, together with ion peaks at 162.03 for amide fragment that indicated loss of the two prenyls from molecular ion. Further structural confirmation of **1** was derived from the calculation of double bond equivalent (DBE) of 8 according to the presence of one cyclic, one carbonyl, and six double bonds, respectively. Based on the analysis of spectral data and compared with published papers, the structure of **1** was determined and proposed as a novel compound isolated from *P. crocatum* and published for the first time in this report. Then, the compound **1** was namely as Piperyamide A or (E)-N-(3,7-dimethylocta-1,6-dien-3-yl)-3-(4-hydroxyphenyl)acrylamide as shown in Figure 1.

#### 2.1.2. Structure Determination of Compound **2**

**Figure 2:**
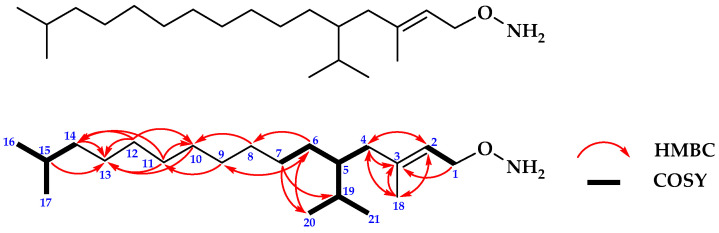
Structure of compound **2** and the HMBC and ^1^H-^1^H COSY correlations.

Compound **2** was isolated as a colorless oil. The ^13^C-NMR and DEPT 135° spectra of **2** revealed twenty-one signals including for five methyls at δc 16.2, 19.8, 19.7, 22.8, and 22.7 ppm; one oxygenated methylene at δc 59.5 ppm; ten methylenes at δc 39.9, 37.5, 25.2, 37.4, 36.7, 29.8, 37.3, 24.5, 24.9, and 39.4 ppm; one olefinic methine at δc 123.1 ppm; three methines at δc 32.8, 32.7, and 28.1 ppm; and one quaternary carbon at δc 140.4 ppm, respectively. Then, the ^1^H-NMR spectrum of **2** showed fifteen protons for five methyls at δ_H_ 1.65 (3H, s), 0.83 (3H, t, J = 3 Hz), 0.83 (3H, t, J = 3 Hz), 0.84 (3H, d, J = 7 Hz), and 0.84 ppm (3H, d, J = 7 Hz). The other proton signals showed ten methylenes at δ_H_ 1.23 (2H, m), 1.36 (2H, m), 1.23 (2H, m), 1.23 (2H, m), 1.12 (2H, m), 1.23 (2H, m), 1.23 (2H, m), 1.05 (2H, m), 1.12 (2H, m), and 1.97 ppm (2H, m), respectively; together with one oxygenated methylene at δ_H_ 4.13 ppm (2H, d, J = 6.5 Hz); three methines at δ_H_ 1.36 (1H, m), 1.36 (1H, m), and 1.50 ppm (1H, m); and one olefinic methine at δ_H_ 5.39 ppm (1H, t, J = 7 Hz) (Table 2), respectively, while of two others proton signals at δ_H_ 1.05 ppm (2H, m). Further, the oxygenated proton signal at C1 was indicated as a hydroxylamine group; C4, C6, C7, C8, C9, C10, C11, C12, C13, and C14, respectively, form a straight chain framework as the main skeleton of compound **2;** C2 and C3 formed a trans double bond position. The suggested main skeleton of **2** was also supported by 2D-NMR analysis of ^1^H-^1^H-COSY and HMBC spectra, of that ^1^H-^1^H-COSY spectrum showed the correlation between oxygenated methylene at H-1 to H-2; methylene proton H-4 to H-5, H-5 to H-6 and H-7; then H-5 to H-19, H-19 to methyls protons H-20 and H-21, together with the correlation of H-14 to H-15, and also to methyl protons H-16 and H-17, respectively. On the other hand, the HMBC spectrum showed the important correlation between H-1 and C3; H-2 and H-3 together connected to C4 and C18; H-4 and C2, C3, and C18; H-6 and C8, C9, and C20; H-7 and C19 and C20; H-8 and C10; H-9 and C6 and C11; H-10 and C13; H-11 and C10 and C 14; H-12 and C10, C13, and C14; H-14 and C13; H-15 and C13; H-18 and C2, C3, and C4; and H-20 and C6, respectively (Figure 2). Based on the 1D and 2D-NMR data, the main skeleton of compound **2** is suggested as a member of an unsaturated and branched long-chain hydroxylamine derivative group [14,15].

The presence of functional groups in compound **2** was identified from IR absorption signals at 3334, 1669, and 1080; 1669 and 1463; 1378 and 1239; 2911; 1463 and 736 cm^−1^, corresponding to primary amine, olefinic carbon, methyl, iso-propyl, and methylene, sequentially, together with data of UV–Vis spectrum which showed an absorption at 203 nm corresponds to the electronic transition of C=C in compound **2**. To confirm the suggested molecular structure of **2** derived from 1D and 2D-NMR data, the mass measurement was conducted and the mass spectrum of compound **2** showed molecular weight (*m*/*z*) was 324.83 (calcd. 325.33 for C_21_H_43_NO). Based on the analysis of spectroscopic spectra and compared to the published paper, the structure of compound **2** was suggested as a novel long and branched chain hydroxylamine derivative group isolated from *P. crocatum* and published for the first time in this report. Then, compound **2** was named as Piperyamine A or (E)-O-(5-isopropyl-3,15-dimethylhexadec-2-en-1-yl)hydroxylamine, as shown in Figure 2.

Based on 1D and 2D-NMR data of compound **2**, the results as shown in Table 2 as follows:

**Table 2:** NMR data of compound **2** (500 MHz for ^1^H and 125 MHz for ^13^C, in CDCl_3_).

No.	^13^C NMR	^1^H NMR
	δc	δ_H_ (Integral, Mult., J = Hz)
1	59.5	4.13 (2H, d, 6.5)
2	123.1	5.39 (1H, t, 7)
3	140.4	-
4	39.9	1.97 (2H, m)
5	32.8	1.36 (1H, m)
6	37.5	1.05 (2H, m)
7	25.2	1.23 (2H, m)
8	37.4	1.23 (2H, m)
9	36.7	1.12 (2H, m)
10	29.8	1.23 (2H, m)
11	37.3	1.23 (2H, m)
12	24.5	1.23 (2H, m)
13	24.9	1.36 (2H, m)
14	39.4	1.12 (2H, m)
15	28.1	1.50 (1H, m)
16	22.7	0.84 (3H, d, 7)
17	22.8	0.84 (3H, d, 7)
18	16.2	1.65 (3H, s)
19	32.7	1.36 (1H, m)
20	19.7	0.83 (3H, t, 3)
21	19.8	0.83 (3H, t, 3)

#### 2.1.3. Structure Determinations of Compound **3**

Compound **3** was isolated as white crystals. The ^13^C-NMR and DEPT 135° spectra of compound **3** showed twenty-nine carbon signals which were assigned as one oxygenated methine at δc 71.9 ppm; two quaternaries at δc 36.2 and 42.3 ppm; one olefinic quaternary at δc 140.8 ppm; two olefinic methines at δc 138.4 and 129.3 ppm; eight methines at δc 121.8, 31.9, 50.2, 56.9, 56.0, 40.6, 51.3, and 31.9 ppm; six methyls at δc 12.1, 19.1, 23.1, 21.2, 19.5, and 12.1 ppm; and nine methylenes carbons at δc 37.3, 31.9, 42.4, 31.7, 21.3, 39.7, 24.4, 29.0, and 25.5 ppm. The ^1^H-NMR spectrum of compound **3** showed the presence of one oxygenated methine proton signal at δ_H_ 3.51 (1H; m) and two olefinic methines at δ_H_ 5.13 (1H, dd, *J* = 8.5; 15 Hz) and 5.00 (1H, dd, *J* = 8.5; 15.4 Hz), while methines protons showed at eight signals at δ_H_ 5.33 (1H, t, 2 Hz), 1.59, 0.9, 1.03, 1.14, 1.23, 1.50, and 1.49 ppm. On the other hand, six methyls were identified at δ_H_ 0.65 (-), 0.99 (-), 1.05 (3H, d, *J* = 7 Hz), 0.78 (3H, d, *J* = 7.5 Hz), 0.83 (3H, d, *J* = 1 Hz), and 0.83 ppm (-). Further, nine methylenes protons at δ_H_ 1.82 (2H, m), 1.59 (2H, m), 2.26 (2H, dd, J = 2; 2 Hz), 1.98 (-), 1.44 (-), 1.28 (-), 1.50 (-), 1.23 (-), and 1.15 ppm (-) were identified. The oxygenated proton signal at δ_H_ 3.51 ppm (1H; m) indicates a hydroxyl group attached to the C-3, while the H-25 proton has an α position for the triterpenoid derivative [16]. The protons signal at δ_H_ 1.1 ppm indicates that compound **3** has a proton on the aliphatic or cyclic frame work of methylene, respectively. The olefinic signals of C-22 and C-23 show a double bond position. The main skeleton of this compound was identified further using the HMBC and ^1^H-^1^H-COSY spectrum. The presence of HMBC signals between H-1 correlated to C3; H-2 to C4; H-4 to C3, C5 and C6; H-6 to C4, C8 and C10; H-7 to C14; H-12 to C13; H-17 to C14; H-18 to C13, C14, C17, and C20; H19 to C1, C5, C9, and C10; H-21 to C13 and C17; H-22 to C3, C21, and C24; H-23 to C24; H-27 to C25; H-29 to C24 and C28, respectively, were analyzed. Further, analysis of the ^1^H-^1^H-COSY spectrum of compound **3** indicated the correlation between protons signals of H-2 at δ_H_ 1.59 ppm (2H, m) to H-3 at δ_H_ 3.51 ppm (1H, m); cyclic signals at C1, C10, C5, and C4; and the OH group was bound to C3 cycloartenol [17]. According to the spectroscopic results, compound **3** is suggested as a steroid derivative attached to a hydroxyl group at C3 and closely related to stigmasterol [18].

**Figure 3:**
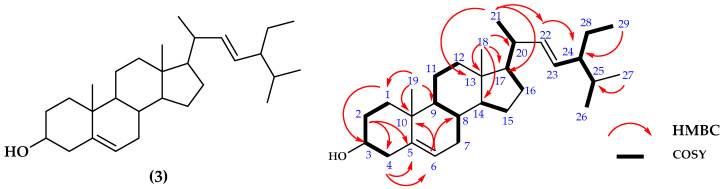
Structure of stigmasterol (**3**) and the HMBC and ^1^H-^1^H COSY correlations.

The IR spectrum of compound **3** showed the absorption signals at 3426, 2868, 1665, 1464, and 1053 cm^−1^ for hydroxyl, methine stretch, olefinic stretch, methine bending, and C-O stretches, sequentially. Aligned with the IR spectrum value, the UV–Vis spectrum of **3** showed that a maximum wavelength peak of 205 nm indicated the presence of an *n*→σ* bond transition and, as a consequence, presented a hydroxyl group of OH-C-C. Further, the molecular weight of this compound was measured to ensure the final structure. From the MS measurement, the identified HR-TOFMS [M+Na]^+^ ion peak at *m*/*z* of compound **3** was 435.3068 (calcd. 412.3608 for C_29_H_48_O), and the result was confirmed by DBE of six for four cyclic and two double bonds. Based on the analysis of spectroscopic data and published papers, compound **3** was identified as triterpenoid’s derivative which is stigmasterol as shown in Figure 3 [19]. 

Based on 1D and 2D NMR data of compound **3**, the results as shown in Table 3 as follows:

### 2.2. Antifungal Assay

The antifungal assay in Table 4 showed that compound **3** has the strongest activity against *C. albicans* ATCC 10231 at all concentrations of 2.5, 5, and 10%, with the highest inhibition zone of 14.5 mm at 10% those close to the inhibition zone value of ketoconazole as a positive control and then followed by compounds **2** and **1**, respectively. Based on the reference, the inhibition zone was strong if more than 6 mm, moderate if in the range of 3–6 mm, and weak if less than 3 mm [20]. Table 4 also showed that compound **3** has the best MIC and MFC at concentrations of 0.31 and 1.2% *w*/*v*, respectively, compared to the other compound in inhibiting *C. albicans* ATCC 10231. Based on the reference, the MIC value is referred to as a strong category if less than 0.01% *w*/*v*, moderate if in a range from 0.01 to 0.0625 % *w*/*v*, and weak if more than 0.0625% *w*/*v* [21]. Compound **3** inhibits the growth in *C. albicans* ATCC 10231 better than compounds **1** and **2**, based on inhibition zone, MIC, and MFC values.

### 2.3. In Silico Assay

**Figure 4:**
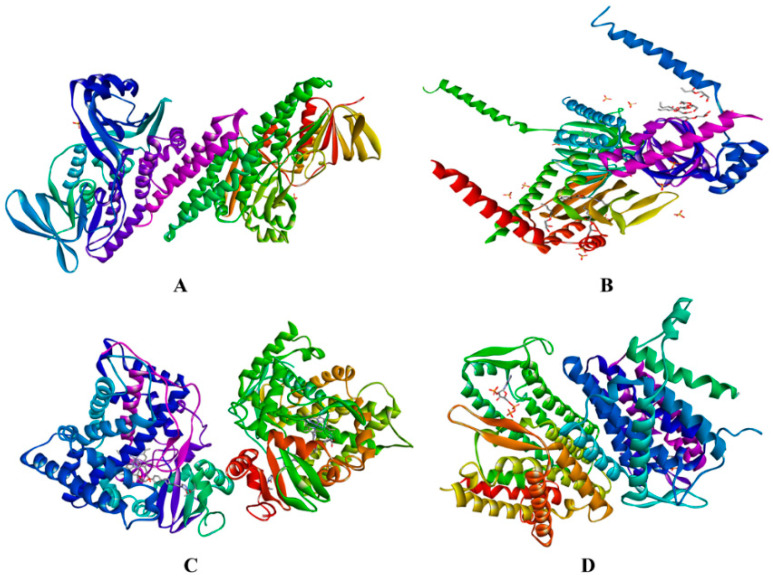
Enzymes of ERG1 (EC.1.14.14.17) (**A**), ERG2 (EC. 5.3.3.5) (**B**), ERG11 (EC.1.14.14.154) (**C**), and ERG24 (EC.1.3.1.70) (**D**) as macromolecules or protein targets in molecular docking.

In this research, an in silico study was used to find out the molecular docking prediction of the ergosterol inhibition mechanism between compounds **1, 2,** and **3** against ERG1, ERG2, ERG11, and ERG24 (Figure 4). 

**Figure 5:**
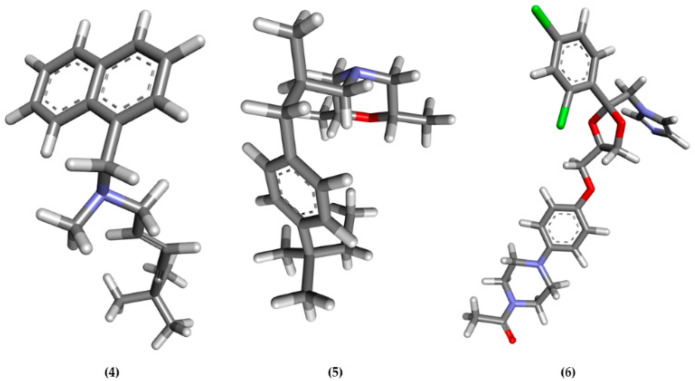
Three-dimensional structure of terbinafine (**4**), amorolfine (**5**), and ketoconazole (**6**) as positive control ligands in molecular docking.

The interaction strength between ligand and protein was analyzed through binding affinity values (ΔG), the inhibition constant (Ki), and intermolecular interactions between compounds **1**, **2**, and **3** compared with the positive control for ERG 1, ERG11, ERG2, and ERG24 used terbinafine (**4**), amorolfine (**5**) and ketoconazole (**6**), sequentially (Figure 5).

As standards in the docking process, the grid box for 6C6N was set at X 14.891, Y 28.085, and Z 60.187; for 5HK1 at X −9.046, Y 20.632, and Z −23.364; for 5FSA at X 188.274, Y 25.121, and Z 74.886; and for 4QUV at X 7.892, Y −0.759 and Z −0.176. Table 5 shows that of all complexes of ligand-ERG1, ligand-ERG2, ligand-ERG11, and ligand-ERG24, ligand **3** has the best binding affinity against all enzymes than the other compounds with ΔG values of −11.14, −12.78, −12.00, and −6.89 Kcal/mol, respectively. Ligand **3** also has the best binding if compared with positive control, either terbinafine at ERG1, ketoconazole at ER11, and amorolfine at ERG2 and ERG24 with values of −7.36, −10.09, −10.99, and −5.25 Kcal/mol, sequentially. It means that ligand **3** has a stronger binding affinity than the positive control, either ligands **1** and **2**. The value of this binding affinity is directly proportional to the inhibition constant (Ki) value, while Ki for ligand **3** was 6.83 × 10^−3^, 4 × 10^−4^, 1.6 × 10^−3^, and 8.88 μM, respectively, smaller than the positive control and ligands **1** and **2**. The smaller the value of ΔG and Ki, the stronger the interaction strength.

**Figure 6:**
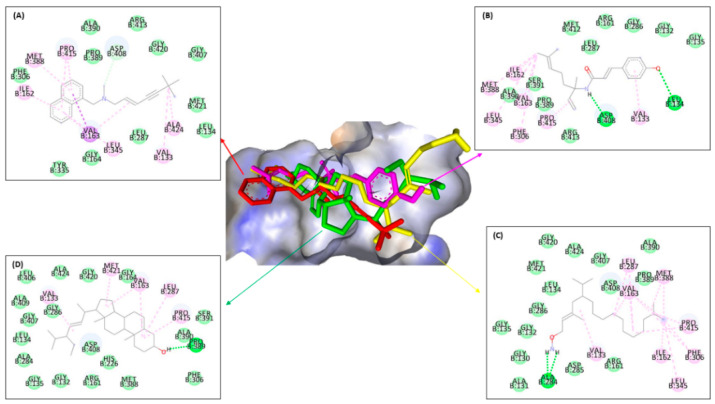
Molecular docking from terbinafine (**A**), ligands **1** (**B**), **2** (**C**), and **3** (**D**) against ERG1.

**Figure 7:**
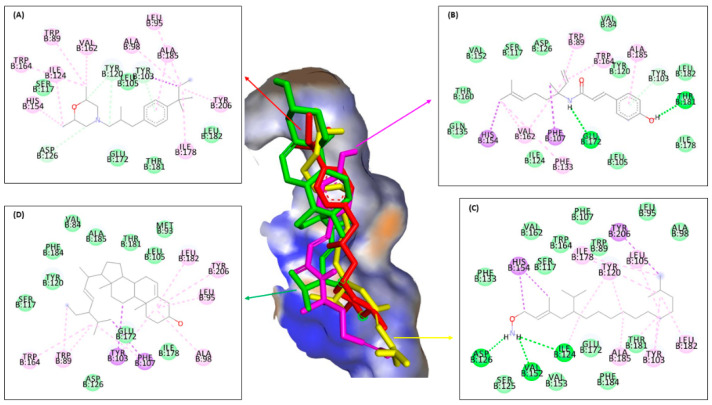
Molecular docking from amorolfine (**A**), ligands **1** (**B**), **2** (**C**), and **3** (**D**) against ERG2.

**Figure 8:**
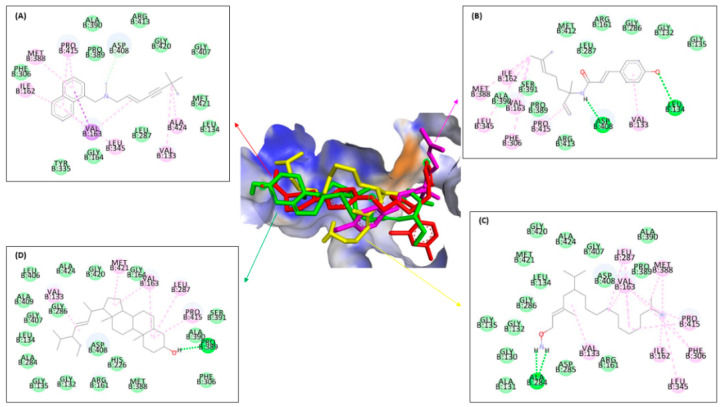
Molecular docking from ketoconazole (**A**), ligands **1** (**B**), **2** (**C**), and **3** (**D**) against ERG11.

**Figure 9:**
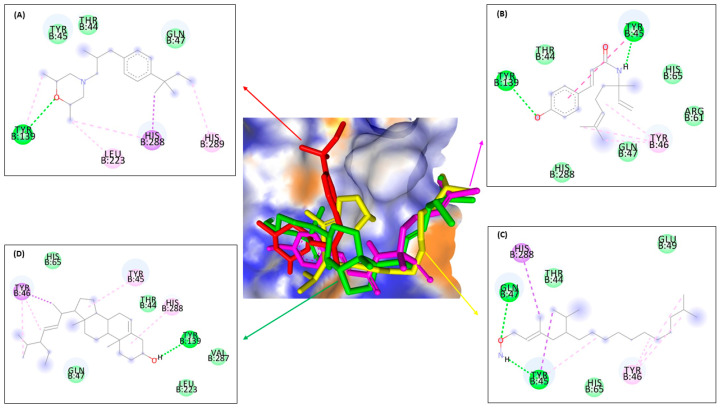
Molecular docking from amorolfine (**A**), ligands **1** (**B**), **2** (**C**), and **3** (**D**) against ERG24.

Further, the study of ergosterol mechanism inhibition using molecular docking was analyzed by intermolecular interactions against four protein targets. The result of molecular docking is shown in Figure 6, Figure 7, Figure 8 and Figure 9 and Table 6. Furthermore, Figure 6 and Table 6 showed that ligand **3** bound the same seven amino acids as positive control, terbinafine. The interactions are seen at valine B:133 in the π–π T-shaped alkyl, and leucine B:134, phenylalanine B:306, alanine B:390, glycine B:164, B:407, and B:420 in the Van der Waals interaction. Differently from ligands **1** and **2**, they only bind residues in the same Van der Waals interaction with the positive control, while ligand **1** bound with alanine B:390, proline B:389, arginine B:413, and leucine B:287; on the other hand, ligand **2** bound with glycine B:420 and B:407, methionine B:421, leucine B:134, alanine B:390, and proline B:389. It means that ligand **3** binds more similar residues in intermolecular interaction to the positive control in the active site against ERG1 more than any other ligands.

Figure 7 and Table 6 indicated almost the same results with ERG1, where ligand **3** bound seven similar residues with amorolfine as the positive control at three interaction types—π–sigma interaction with tyrosine B:103, in π–π T-shaped alkyl with tryptophan B:89 and B:164, and in Van der Waals interaction with serine B:117, threonine B:181, leucine B:105, and glutamine acid B:172. Ligand **1** binding six residues in the same interaction with amorolfine at four different interactions, namely tyrosine B:103 in π–donor hydrogen bond interaction; valine B:162 in π–π T-shaped alkyl; alanine B:185 in π–alkyl and serine B:117; and leucine B:182 and 105 in Van der Waals interactions, while ligand **2** only binds in two same interaction types, π–π T-shaped alkyl with isoleucine B:124 and Van der Waals with serine B:117, threonine B:181, leucine B:105, and glutamine acid B:172. Ligand **3** binds more residues in different types of intermolecular interaction with amorolfine as the positive control against ERG2 in the pocket binding site.

Figure 8 and Table 6 showed almost the same results as the previous enzyme. Ligand **3** binds thirteen residues with the same amino acid with ketoconazole against ERG11 of leucine B:376 at π–alkyl; at Van der Waals binding phenylalanine B:233, B:380, and B:228, proline B:230, serine B:378 and B:507, histidine B:310, threonine B:311, leucine B:121, glycine B:65, tyrosine B:505, and serine B:506; and ligand **2** binds the same amino acid with methionine B:508 at π–π T-shaped alkyl; proline B:230, phenylalanine B:126, glycine B:307, and leucine B:121 at Van der Waals interaction types with the positive control ketoconazole. On the other hand, ligand **1** only binds the same residue type at Van der Waals interactions with glycine B:65 and 307, proline B:230, serine B:378, phenylalanine B:228 and B:126, histidine B:310, and glycine B:307. It can be concluded that ligand **3** binds more amino acids than ligands **1** and **2**, similar to positive control against ERG11, either to ERG1 or ERG2.

Figure 9 and Table 6 show that the bonds that occur in all ligands against ERG24 tend to be less than the bonds in the previous enzyme, such as ERG1, ERG2, and ERG11. Ligand **3** had a similar interaction binding with amorolfine as the positive control at two interaction types, namely π–alkyl with histidine B:288 and at Van der Waals with threonine B:44 and glutamine B:47. Ligand **1** only bound three residues at two interactions, conventional with tyrosine B:139, and Van der Waals with threonine B:44 and glutamine B:47. Different with ligand **3**, while only binding two amino acids in two-similar interaction type with amorolfine at π–sigma with histidine B:288, and threonine B:44 at Van der Waals interaction against ERG24. Ligand **3** still has the most types and number of bonds when compared to the other two ligands, similar to all positive controls against ERG.

### 2.4. ADMET and Drug-Likeness Analysis

ADMET consists of absorption, distribution, metabolism, excretion, and toxicity, as shown in Table 7.

Absorption prediction consists of water solubility, intestinal absorption, and skin permeability parameters. Table 7 showed that the water solubility for compounds **1**, **2,** and **3** were −4.674, −7.66, and −6.673 log mol/L, respectively. This means that these three compounds have good water solubility in the body because the best category values of water solubility are less than 0, and the best value is less than −0.5 [23]. These three compounds also have good intestinal absorption with values of more than 80% [24], 89.583%, 90.281%, and 96.151% absorbed, respectively, while compound **3** has the best absorption in the intestinal. These three compounds also have the non-sensitizer category for skin permeability, with −2.7, −2.792, and −2.781 log Kp, respectively.

The parameters of drug distribution covered volume distribution (VDss), the blood–brain barrier (BBB), and central nervous system (CNS) permeability. Table 7 showed that these three compounds were poor in the blood’s drug distribution, with 0.366, 0.11, and 0.18 log L/kg, while good VDss ranged from 0.5 to 3 L/Kg [25]. To determine drug distribution using BBB and CNS permeability parameters, criteria consist of high absorption (>2.0), moderate absorption (0.1–2.0), and low absorption (<0.1) [26]. Compounds **1** and **2** had low absorption with −0.062 and −0.414 log BB, while compound **3** had moderate absorption with 0.799 log BB. These three compounds also had low absorption at CNS permeability, with −2.12, −1.36, and −1.737 log PS, respectively. Compounds **1** and **2** were predicted to have difficulty entering BBB and CNS systems, and compound **3** had difficulty penetrating the CNS system, which means that these compounds were assumed to be safe for the brain.

The prediction of drug metabolism is also influenced by enzyme Cytochrome 450 (CYP) inhibition [27]. Compound **3** does not inhibit any enzymes of CYP1A2, CYP2C19, CYP2C9, CYP2D6, and CYP3A4. Compound **2** only inhibits CYP1A2, and compound **1** inhibits CYP1A2, CYP2C19, and CYP2C9. It can be predicted that the digestive system will not be affected by compound **3**.

Further, the excretion parameter is shown from total clearance. Table 7 shows that compound **2** has the fastest molecule excretion with 1.861 log ml/min/kg, which means it has a better effect than other compounds. The higher the total clearance value, the faster the drug excretion process that occurs in the body [28]. The last parameter from pharmacokinetic properties was acute oral toxicity shown by the lethal dose 50 (LD_50_), a statistical parameter that estimates the number of deaths in 50% of animals when given several drugs as a single dose at a certain time [29,30,31]. Table 7 showed that compound **2** was in the category may be harmful if swallowed, with an LD_50_ of 1.654 mol/kg, while compounds **1** and **3** were in the harmful if swallowed category with an LD_50_ of 2.331 and 2.375 mol/kg, respectively. Both compounds **1** and **3** have non-skin sensitization, different from compound **2,** which has a skin sensitizer.

The drug-likeness analysis of this research followed Lipinski’s Rule of Five (Ro5) with five main parameters, by molecular mass (<500 daltons), hydrogen bond donors/HBD (<5), hydrogen bond acceptors/HBA (<10), octanol/water partition coefficient/log *p* (<5), and molecular refractivity (40–130) [32,33]. Table 8 shows that these three ligands (compounds **1**–**3**) have molecular weights below 500 daltons, with 299.41, 325.57, and 412.69 daltons, and have hydrogen bond donors less than 5, with 2, 1, and 1, respectively. These three ligands have hydrogen bond acceptors less than 10 with 3, 2, and 1, respectively. Ligand **1** has a logP of less than 5, at 4.6, while ligands **2** and **3** have logP values of 7.35 and 7.8, sequentially. Ligands **1** and **2** have molecular refractivity ranging from 40 to 130 of 93.52 and 106.38, while ligand **3** has 132.76. Ligands **1** and **2** can be assumed to be used as oral drugs because they have zero violations and one violation, respectively, while ligand **3** has low solubility and permeability because it has two violations in the Rule of Five [34].

## 3. Discussion

*C. albicans* is one of the opportunistic fungal pathogens on human healthy mucosal surfaces that normally occurs in 50% of the population worldwide [32]. The decreased body resistance causes the colony of *C. albicans* to increase in the body and causes various infections such as candidiasis [33]. Currently, the discovery of new prospective natural anti-fungal agents that have selective, specific, effective, low cost, low side effects, and are appropriate to treat fungal infected targets, has become an interesting research focus to resolve some infectious diseases due to *C. albicans*. Further, the antifungal discovery studied some natural medicinal plants using bioactivity-guided purification of lead compounds as an anti-fungal constituent. Based on the ethnobotany and ethnopharmacology data, the edible herbal plant *P. crocatum* was selected as a source to isolate new antifungal agents against *C. albicans* [35]. In the previous report, the extracts and active compounds of this plant were active with the following properties: antibacterial [36], antimicrobial [37], anti-cancer, anti-oxidant [38], antibiotics [30], antihyperglycemic [31], anti-inflammation [39], analgesic, immunomodulator, anti-tumor, and anti-fungal [40,41], respectively.

Separation and purification of anti-fungal constituents of *P. crocatum* extract were used as bioactivity assays against *C. albicans* ATCC 10231 leading to the isolation of anti-fungal compounds. Further, the structures were identified as new anti-fungal of Piperyamide A (**1**) and Piperyamine A (**2**) together with stigmasterol (**3**), respectively. The structures are shown in Figure 1, Figure 2 and Figure 3. The previously published paper showed that the extract of *P. crocatum* was active against *C. albicans* non-ATCC [42], while the active constituent as an anti-fungal agent against *C. albicans* non-ATCC was reported for estragole [43,44], hydroxychavicol [45], and carvacrol [46].

Two new compounds of Piperyamide A (**1**) and Piperyamine A (**2**) and their activity as anti-fungal against *C. albicans* ATCC 10231 were published for the first time in this paper. Stigmasterol (**3**) is well known as an important natural small molecule that has been used as a drug for some diseases, and is also reported as active as an anti-fungal against *C. albicans* ATCC 10231 [47,48], *Candida parapsilosis* ATCC 220199 [49], *Macrophomina phaseolina* [50], *Saccharomyces cerevisiae* [51], *Aspergilus fumigatus* [52], and other bioactivities [53,54]. Further, the procedure of isolation, characterization, and identification of stigmasterol (**3**) also was reported in some published papers [55,56]. Based on the anti-fungal assay against *C. albicans* ATCC 10231, stigmasterol (**3**) was the most active compared to **1** and **2** with zone inhibition values of 14.5, 11.9, and 13.0 mm, respectively, at a concentration of 10% *w*/*v*, together with MIC/MFC at 0.31/1.2% *w*/*v*.

It can be suggested that compounds **1**–**3** have potential as new prospective candidate anti-fungal agents against *C. albicans* ATCC 10231. Some natural anti-fungal bioactive compounds against *C. albicans* ATCC 10231 were reported in some papers [57,58,59], but the anti-fungal activity of compounds **1**–**2** against *C. albicans* ATCC 10231 was reported for the first time in this research. This study adds to the list of natural anti-fungal compounds against *C. albicans* ATCC 10231. Compound **3,** known as stigmasterol, has the best antifungal activity against *C. albicans* ATCC 10231 compared to compounds **1** and **2**. Based on the literature, stigmasterol from *Neocarya macrophylla* has antifungal activities against *C. albicans* and *Candida krusei* with MIC values of 6.25–25 μg/mL [50]. The stigmasterol also inhibits two other *Candida* species, *Candida tropicalis* and *Candida virusei* with MIC 50 and 12.5 μg/mL [55]. Stigmasterol and β-sitosterol from *Alpinia conchigera* against *C. albicans* ATCC 10231 showed MIC value at a concentration of more than 0.25%, while stigmasterol from *Uvaria scheffleri* showed zone inhibition of 1.6 ± 2.0 mm, and from *Icacina trichantha* showed zone inhibition of 21 mm at 50 μg/mL against *C. albicans* [60,61,62]. One of the differences in the results of this antifungal activity is caused by the difference in the type of *C. albicans* used, between ATCC and non-ATCC.

For the breakpoint of azoles as the positive control, declared to be in the resistant (R) category, when the value is ≤14 mm, the susceptible-dose dependent (S-DD) range is from 15 to 18 mm, and it is susceptible (S) if it is ≥19 mm against *C. parapsilosis* ATCC 22019, *C. krusei* ATCC 6258, *C. albicans* ATCC 90028, and *C. tropicalis* ATCC 750, while the zone diameters at *C. albicans* ATCC 90028 for fluconazole, voriconazole, posaconazole, and caspofungin are 28–39, 31–42, 24–34, and 18–27 mm, respectively [63]. Based on Table 4, ketoconazole, as the positive control, has zone inhibition of 30.0, 31.2, and 32.2 mm at concentrations of 2.5, 5, and 10% *w*/*v*, respectively, which means ketoconazole is a susceptible drug against *C. albicans*. This azoles group defines lanosterol 14α demethylase (ERG11) from ergosterol biosynthesis as a target protein against *C. albicans*, while the allylamines and thiocarbamates groups focused on squalane epoxidase (ERG1), the morpholines group on sterol 14Δ reductase (ERG24), the hydroxyanilides or amino-pyrazolinones group targeted on C-4 sterol methyl oxidase (ERG25), the C-4 sterol decarboxylase (ERG26), C-4 sterol ketoreductase (ERG27), and morpholines group focused on sterol 8Δ isomerase (ERG2), and the polyene macrolides group on C-24 sterol reductase (ERG4) in antifungal mechanisms [59,60]. Ergosterol is located along the *C. albicans* plasma membrane lipid bilayer consisting of bitopian endoplasmic reticulum proteins, which are coated by chitin, β-(1,3)-glucan, β-(1,6)-glucan, and mannoproteins which form galactosaminoglycan [64,65,66]. Ergosterol is a main enzyme in *C. albicans* sterol, which functions to maintain fungal cell membrane permeability and integrity, causing a fungistatic effect and, nowadays, is an important target site for antifungal drug invention [67,68].

Further, molecular docking is needed to predict the active site of a protein and analyze the interaction between the ligand and the target protein [69]. Based on Table 5, it was seen that ligand 3 has the best binding affinity than the other ligands. The predicted protein–ligand binding occurs as an impact of change in binding affinity (ΔG), the negative free energy spontaneously [64], which is an important to lead compounds being drug candidates in molecular docking because of the receptor stability [65,66]. To predict docked protein–ligands orientation and binding affinity in the active site, the hydrogen bonds and docking score for determination of the biomolecule structure and function are required [70,71]. Whereas constant inhibition (Ki) signifies a parameter at half-maximum concentration to make an inhibitor effect [72,73]. Also, ligand **3** has the best Ki value than positive control, ligands **1**, and **2** against all targetted enzymes. The result indicated that ligand **3** is more effective for enzyme inhibition against ERG1, ERG2, ERG11, and ERG24 than the positive control and the other compounds based on their ΔG and Ki. On the other hand, compound **1** showed a binding affinity and Ki value almost close to positive control. By molecular docking, compounds **3** and **1** are claimed as new candidate compounds.

Figure 6, Figure 7, Figure 8 and Figure 9 and Table 6 show that ligand **3** binds more to similar amino acids and has more of an intermolecular interaction type with the positive control, either terbinafine at ERG1, ketoconazole at ERG11, amorolfine at ERG2 and ERG24, than ligands **1** or **2**. Compound **3** binds at two interaction types in π–π T-shaped alkyl and Van der Waals interactions with seven residues binding against ERG1. Against ERG2, compound **3** binds with three interaction typesπ–sigma interaction, π–π T-shaped alkyl interaction, and Van der Waals interaction. At ERG11, compound **3** binds thirteen residues at π–alkyl, Van der Waals, and π–π T-shaped alkyl, while the bonds that occur in all ligands against ERG24 tend to be less than the bonds in the ERG1, ERG2, and ERG11. Van der Waals is the most common interaction from all ligands that has a major role in hydrogen bond formation and Coulombic interactions [74]. A hydrogen bond is known as the master key of molecular recognition that is stronger than Van der Waals and weaker than covalent bonds and has an important role in mediating drug receptor binding and molecule physicochemical properties [70]. Based on this, compound **3** still has the most types and number of bonds when compared to compounds **1** and **2**, similar to each positive control against all enzymes.

Based on the structureactivity relationship (SAR), compound **3** has a different structure from compounds **1** and **2**. Compound **3** is structurally characterized by a side-chain having ten carbon atoms that are connected at C-17 of the steroid skeleton, a hydroxyl (OH) group attached to C-3 of the benzene ring position, and two methyl groups at the C-25, C-28, C-20, and C-10 positions as substituents. Compound **1** has a hydroxyl group at the C-4′ position on the benzene ring; an amides group at the C-2 and C-1″ positions; two methyl groups at the C-5″ and C-1″ positions of the steroid skeleton, while compound **2** has an amines group at C-1, each of the two methyl groups at the C-15 and C-19 positions, as well as at the C-3 position of the steroid skeleton. This confirmed the importance of the hydroxyl (OH) group in compounds **3** and **1** as an antifungal-activity-increasing factor, which is not possessed by compound **2**. The Hydroxyl group has a significant effect on antifungals via its effect on fungal cell cytotoxicity [75,76]. Since the presence of hydroxyl groups and this delocalized electron (double bonds) system plays a crucial role in the antimicrobial activity as a proton exchanger, lowering the gradient across the cytoplasmic membrane ultimately results in collapse of the proton motive force and depletion of the ATP pool ultimately leading to cell death, and the hydroxyl group is thought to have a stronger binding ability than the methoxy group [77,78]. These three compounds also have the same functional group—the methyl group. The methyl group was extremely active against yeast but ineffective against bacteria [79].

**Figure 10:**
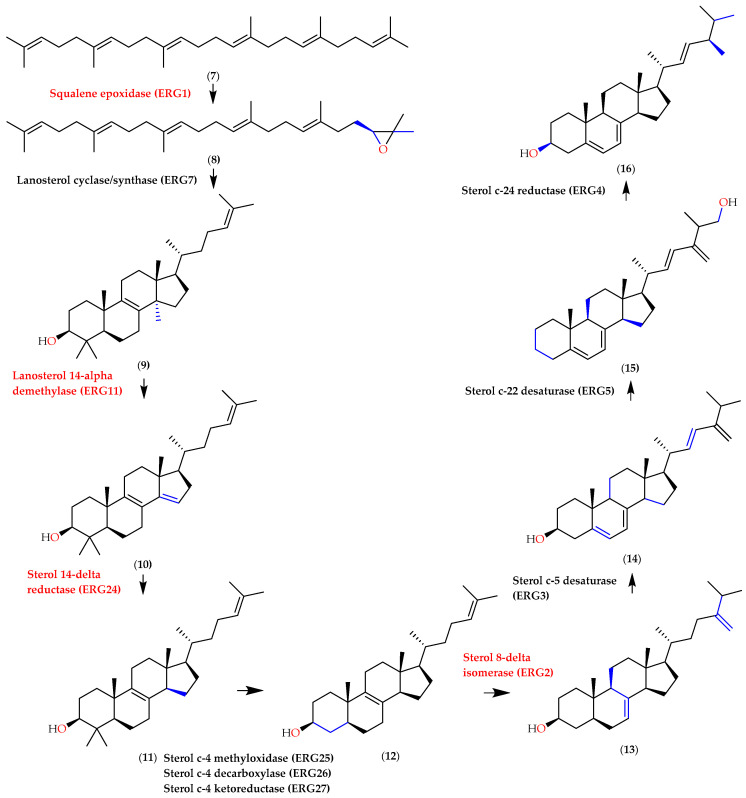
Ergosterol pathways in fungal, with squalene (**7**), squalene 2,3-epoxidase (**8**), lanosterol (**9**), 4,4-Dimethylcholestas-8,12,24-trienol (**10**), 4,4-Dimethylzymosterol (**11**), zymosterol (**12**), episterol (**13**), ergosta-5,7,24 (28)trienol (**14**), ergosta-5,7,22,24 (28)tetraenol (**15**), and ergosterol (**16**) with the affected enzymes [82].

Several antifungal groups work by inhibiting ergosterol biosynthesis (Figure 10), a neutral lipid of the fungal membrane. ERG1 (squalene epoxidase) is an antifungal targetted of allylamines or thiocarbamates, while ERG2 (C_8_-C_7_ sterol isomerase) is a fungal gene encoding that also has an important thing of ergosterol pathways [80]. ERG2 and ERG24 genes in *C. albicans* were blocked by the group of morpholines. ERG11 (lanosterol 14α demethylase) in the ergosterol biosynthesis pathway was inhibited by azoles, the largest antifungal agents. The inhibition of 14α demethylase will cause ergosterol to be depleted and changed to another sterol, causing the change in permeability and fluidity of the fungal cell membrane and becoming brittle [81].

The discovery of pharmaceutical drugs is an expensive process and needs a long time [83,84]. As a requirement for an Investigational New Drug (IND) application, the sponsor requires proof of the safety of the candidate molecule [85]. To ensure the safety of the candidate molecules after in vitro and molecular docking analyses, possible drug candidate inventions underwent ADMET and drug-likeness analyses. ADMET functions to identify the pharmacokinetic properties of a compound or drug to determine its function in the body [86,87]. The use of *P. crocatum* as an alternative antifungal treatment provides an opportunity for natural products that can be used as new antifungal drug candidates by first knowing the safety of the candidate molecule using ADMET predictions to minimize failures that may occur during clinical trials, as well as increasing efficiency drug development. To analyze ADMET, we used PkCSM as a new method to predict the drugs’ pharmacokinetic and toxicity properties from graph signatures based on distance [88]. Based on the reference, stigmasterol (**3**) has no liver and hepatic toxicity, is used in animals safely, has high binding stability from the target protein, and has high blood–brain barrier (BBB) permeability [89,90].

Water solubility is a key parameter of the pharmacokinetic properties process. Drug bioavailability was categorized as good if the water solubility value was less than 0, and the best value was less than −0.5 [23]. Table 7 shows that compounds **1**, **2**, and **3** have the best water solubility in the absorption process, good intestinal absorption, and are non-sensitizers at skin permeability. The value range for non-sensitizers is from −3.05–(−1.6) log Kp, whereas sensitizers range from −3.62–(−1.28) log Kp [91]. These three compounds have good absorption parameters (water solubility less than −0.5, intestinal absorption more than 80%, and non-sensitizer skin permeability). 

Table 7 showed that compounds **1** and **2** had low absorption, while compound **3** had moderate absorption of BBB permeability. These three compounds also had low absorption at the central nervous system (CNS) permeability, so these compounds were assumed safe for the brain because they were unable to pass BBB properly. The blood–brain barrier (BBB) is one of the barriers that separate the peripheral nervous system (PNS) and CNS and consists of capillary endothelial cells that are very dense in brain tissue and function to separate interstitial fluid and oxygenated blood, nutrition, and hormones. It protects the brain from chemicals contained in the blood, and maintains the homeostasis of the brain’s microenvironment [92,93,94].

Compound **3** does not inhibit any CYP enzymes in the digestive system as shown in Table 7, while compound **1** only inhibits one enzyme in digestion, CYP1A2, while compound **2** inhibits CYP1A2, CYP2C19, and CYP2C9 enzymes. The CYP enzyme is an important enzyme in the digestive system and has a function in the first phase of the metabolic process of the intestines [27]. The total clearance value is identical to the molecule excretion, while the higher the value of total clearance, the faster the excretion process of the molecule which means it will have a good effect on the body [28].

Based on Table 7, compounds **1** and **3** have no skin sensitizer impact, different from compound **2**. This is influenced by four main biological parameters, such as the time-dose exposure, binding of target protein, variation in each individual, and the activation of dendritic cells [93,94] because skin permeability is one of the chemical parameter factors that affect skin sensitization [95]. Skin sensitization is caused by contact and penetration of chemicals into the skin, which is initiated by molecular haptenation proteins, thus triggering allergic contact dermatitis (ACD), which is a slow-type hypersensitivity condition due to the biotransformation of changes in the allergen potential [91,96,97]. Lipophilic small molecules (under 500 daltons) have a good ability to pass through the stratum corneum membrane and bind to nucleophilic residues in skin proteins, thereby forming stable conjugates, namely hapten–protein complexes that trigger T cell proliferation in naïve T cells and enter into the blood vessels from the lymph nodes via the thoracic duct [98,99,100].

The analysis of LD_50_ in this study used PROTOX, which functions to predict the toxicity of a compound or molecule that is consumed orally in rats as a drug, with a unit value of mg/kg body weight. LD_50_ is the lethal dose when a given dose of the drug will result in 50% mortality in subjects exposed to the drug [101]. Based on Table 8, compound **2** may be harmful if swallowed, while compounds **1** and **3** were in the criteria of harmful if swallowed, with 2.331 and 2.375 mol/kg, respectively. These criteria follow the Globally Harmonized System (GHS), where class I is fatal if swallowed (LD_50_ ≤ 5 mg/kg), class II is fatal if swallowed (5 < LD_50_ ≤ 50 mg/kg), class III is toxic if swallowed (50 < LD_50_ ≤ 300 mg/kg), class IV is harmful if swallowed (300 < LD_50_ ≤ 2000 mg/kg), class V is may be harmful if swallowed (2000 < LD_50_ ≤ 5000 mg/kg) and class VI is non toxic (LD_50_ > 5000 mg/kg) [100,102].

To determine if the compound can be considered as a potential drug, Lipinski’s Rule of Five (Ro5) was used with the criterion being no more than one Ro5 violation [103]. Ro5 dictates that drug molecules must be able to be absorbed properly by the gastrointestinal system in the human intestine, have the ability to penetrate membranes with good bioavailability, have a molecular weight of less than 500 daltons, have a logP less than 5, an HBD less than 5, and an HBA less than 10 [104]. This method also can be used to predict the probability of success of a compound to be developed as an active drug by considering its biological and pharmacological activities [105]. Compounds **1**, **2**, and **3** are examined regarding whether they meet Lipinski’s Ro5 criteria; then, we predict whether there are similarities between these compounds and drugs and if they can be used as potential active drugs that can be consumed orally by humans. Based on Table 8, compounds **1** and **2** met the criteria of Ro5 because they only had one violation or lesss, so they can be used as an oral drug, while compound **3** shows low permeability and solubility as an oral drug.

## 4. Materials and Methods

### 4.1. Material

#### 4.1.1. Plant Material

**Figure 11:**
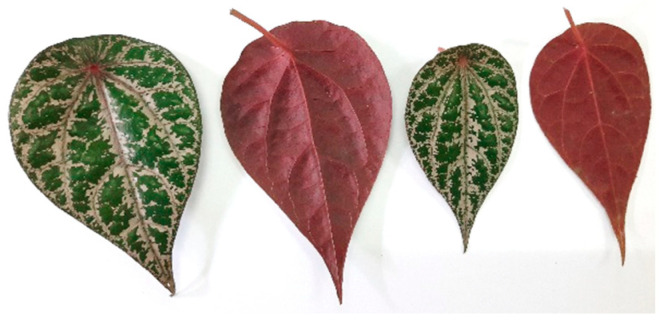
*P. crocatum* leaves (personal collection).

Matured Red Betel leaves (*P. crocatum)* (Figure 11) were collected in November 2021 from a local forest in Sambas, West Kalimantan, Indonesia. The plant was identified at the Laboratory of Biosystematics and Molecular, Department of Biology, Faculty of Mathematics and Natural Sciences, Universitas Padjadjaran, Indonesia.

#### 4.1.2. In Vitro Assay Materials

The chemicals for the extraction and purification used organic solvents distillate methanol, *n*-hexane, ethyl acetate, chloroform, and aqua dest (Ikapharmindo Putramas, Jakarta, Indonesia). The purification by column chromatography used Silica ODS RP-18 (0.040–0.063 mm, Merck, Rahway, NJ, USA) and Silica G 60 (0.063–0.200 mm, Merck). Silica G 60 F_254_ and ODS RP-18 F_254_S (KGaA-made in Germany) with 10% H_2_SO_4_ (*v*/*v*) in ethanol for visual chemical identification analysis used on the thin-layer chromatography (TLC). The antifungal test used an anaerobic jar for the antifungal assay (Merck Co., Ltd., Guangzhou, China and Sigma Aldrich), *C. albicans* with ATCC 10231 (R4601503) Liofilchem (PT Fadhil Damar Putra, Banten, Indonesia) was used as the tested fungal, a ketoconazole tablet 200 mg (Kalbe company) was used as the positive control, methanol and sterile water for injection (PT Ikapharmindo Putramas) were used as the negative control, Himedia brand Potato Dextrose Agar (PDA) dan Himedia Potato Dextrose Broth (PDB) GM403–500G was used as the fungal medium, a Petri dish was used for susceptibility testing (Sigma Aldrich, St. Louis, MO, USA—Whatman, diameter 4 mm), a 96-well microplate was used (Iwaki, Asahi Glass Co., Ltd., Tokyo, Japan), 3820024), 1% Barium chloride (BaCl), 1% H_2_SO_4_, and NaCl physiological sodium chloride (Otsuka, Tokyo, Japan).

#### 4.1.3. In Silico Assay Materials

The material used a 3D structure of squalene epoxidase (ID: 6C6N), lanosterol 14α demethylase (ID: 5V5Z), sterol 14Δ reductase (ID: 4QUV), and sterol 8Δ isomerase (ID: 5HK1) from the Protein Data Bank (PDB) using the RSCB Programs (https://www.rscb.org) with 6C6N, 5V5Z, 4QUV, and 5HK1 format PDB [106,107]. The receptor used a protein squalene epoxidase with UniprotKB-P32476, Cytochrome P-450 lanosterol 14α-demethylase with UniprotKB-C7SEV3, sterol 14Δ reductase with UniprotKB-P32462, and sterol 8Δ isomerase with UniprotKB-P32352 from UniProt knowledgebase (http://www.uniprot.org/) [108]. 

The native ligands are FAD (flavin-adenine dinucleotide), 5FSA (posaconazole), NDP (NADPH dihydro-nicotinamide-adenine-dinucleotide phosphate), and sigma non-opioid intracellular receptor. The tested ligands are compounds **1**–**3**. The compounds were retrieved from PubChem with ID compound **3** 5280794, while terbinafine (ID 1549008), ketoconazole (ID 456201), and amorolfine (ID 54260) were used as positive control ligands. All these data were taken from the PubChem compound database (https://www.ncbi.nlm.nih.gov/pccompound, accessed on 1 June 2023) [109].

#### 4.1.4. ADMET and Drug-Likeness Analysis

A (absorption), D (distribution), M (metabolism), and E (excretion) were used to analyze the pharmacodynamics of the tested and selected compounds [110]. The chemical ligands notation was copied from PubChem in the form of canonical SMILES (https://pubchem.ncbi.nlm.nih.gov/) accessed on 1 June 2023), while T (toxicity) was used to predict ligands toxicity. 

#### 4.1.5. Instruments

The structure of active compounds of *P. crocatum* was determined by spectroscopic analysis of 1D and 2D-NMR, IR, UV–Vis, and mass spectrometry. To visualize, we used TLC plates with UV detector lamps with wavelengths of λ_max_ at 254 and 365 mm. For antifungal activity assay, we used micropipettes, microtubes, incubators, laminar airflow, autoclave machine HVE-50 Hirayama, and paper discs.

### 4.2. Methods

#### 4.2.1. Preparation of Extracts and Compounds

The fresh leaf of *P. crocatum* (5 kg) was cut into small sizes and extracted with methanol 50 L three times over 24 h and then evaporated in a rotary evaporator at ±40 °C to obtain the result of crude methanol extract (100 g). Further separation of the extract by chromatographed on Silica G 60 eluted with *n*-hexane-EtOAc of 10%. The active fraction of 4.1 was chromatographed on ODS RP-18 eluted with MeOH-H_2_O of 10 and 2.5% stepwise, and then purified by repeated chromatography on Silica G 60 eluted with *n*-hexane-EtOAc of 10%, *n*-hexane-CHCl of 5% stepwise, respectively, producing compound **1**. Further purification of active fraction 4.2 by repeated chromatographed on ODS RP-18 eluted with MeOH-H_2_O of 10% stepwise and on Silica G 60 eluted with *n*-hexane-EtOAc of 0.5%, stepwise, gave compound **2**. Purification of active fraction 4.3 by washing out the resulting solid by *n*-hexane gave compound **2**.

#### 4.2.2. In Vitro Assay

##### Structure Determination of Compounds **1**–**3**

The structure of compounds **1**–**3** was determined using a comprehensive analysis of their spectroscopic data of 1D and 2D-NMR (500 MHz for ^1^H and 125 MHz for ^13^C, in CDCl_3_), UV–Vis, IR, and MS. 

##### Antifungal Assay

The antifungal activity of extracts and compounds against *C. albicans* ATCC 10231 was assayed using the Kirby–Bauer disk diffusion method, according to CLSI protocols as the standard assay procedure [111]. The fungus was cultured by growing 1 dose in 5 mL slanted agar solution (Potato Dextrose Agar/PDA) in a zig-zag way and incubated for 48 h at 35 °C. After the incubation process, 1 dose of *C. albicans* culture was inserted into 5 mL physiological NaCl. The optical density of the solution was measured using a microplate reader at 620 nm and diluted by physiological NaCl until equivalent to Mc. Farland 0.5 fungal solution. This fungal solution (100 μL) was added by a spread bar into a Petri dish that contained 5 mL PDA. The methanol compound solution was varied in the series concentration of 2.5, 5, and 10%. In total, 20 μL of the compounds, ketoconazole (positive control), methanol, and sterile water (negative control) was dripped on the paper disc (6 mm) surface on PDA containing *C. albicans*. Further, the samples were incubated for 24 h at 37 °C [112]. After 24 h, the inhibition zone values (mm) were measured.

The method used to measure the MIC and MFC against *C. albicans* was the micro-dilution method. The medium used Potato Dextrose Broth (PDB) to culture *C. albicans*, and was put into a 96-well microplate. In total, 100 μL of the compounds was added to the microplate and then the concentration was diluted gradually. The 0.5 Mc Farland fungal solution was measured in a microplate reader at 620 nm, and then 5 μL was added, respectively, to the microplate and incubated at 37 °C. After 24 h, the microplate was measured in a microplate reader to identify the optical density [73]. The observed optical density value was used to identify the minimum concentration of samples to inhibit the fungal by the MIC value [113]. To analyze the MFC value, the measured diluted concentration in the microplate was spread on the PDA medium and incubated for 24 h at 37 °C.

#### 4.2.3. In Silico Assay

The four ligands, namely FAD (flavin-adenine dinucleotide), 61W sigma non-opioid intracellular receptor, 5FSA (posaconazole), and NDP (NADPH dihydro-nicotinamide-adenine-dinucleotide phosphate), were identified as native ligands. Compounds **1**–**3** and the three positive controls (terbinafine, amorolfine, and ketoconazole) were used as the tested ligand to inhibit ERG1, ERG2, ERG11, and ERG24 in ergosterol biosynthesis. These ligand candidate structures were taken from PubChem (https://pubchem.ncbi.nlm.nih.gov/) accessed on 1 June 2023 and NMR-analyzed results. Further, these chemical structures were converted using Canonical SMILES into 3D (ID compound for compound **3**: 5280794; terbinafine: 1549008; amorolfine: 54260; ketoconazole: 456201; FAD: 643975; sigma non-opioid intracellular receptor: 144418; 5FSA: 468595; and NDP: 830222) using OPEN BABEL Program 2.4.2. in PDB format, whereas the ERG1, ERG2, ERG11, and ERG24 3D structures were drawn from the RSCB program with the 6C6N, 5HK1, 5V5Z, and 4QUV format PDB, sequentially [106].

Autodock tools-1.5.6 was used to analyze the ligand–protein interaction of molecular docking, and the open-source PyRx 0.8 software was used to identify the virtual screening [114]. The first step in using Autodock tools is to prepare a 3D protein structure from a protein data bank (PDB format), process the structure by removing water molecules, stabilize the charge, fill in missing residues, and activate side chains according to the available parameters. The next step is selecting the active site of the receptor and removing water molecules and hetero atoms. Further, the preparation of ligands was carried out with a Chem sketch with a molecular mass of less than 500 Da. The final step in this stage was to analyze the ligand interactions attached to the protein and select a score from the best ligand complex. Compounds **1**, **2**, and **3** as the test ligands were used to bind the protein targets ERG1, ERG, ERG11, and ERG24, for which the ligands were free to blind dock. The lowest bond energy conformation with a root-mean-square deviation (RMSD) value less than 1.0 Å score was the most favorite item.

The Discovery Studio Biovia with 3D molecules was applied to the docking. To analyze the ligand–protein interaction and docking position, an online web server at https://proteins.plus/ was used. Furthermore, the ligand positions of compounds **1**–**3** on enzymes ERG1, ERG2, ERG11, and ERG24 compared to the positive control position on the enzymes were analyzed.

#### 4.2.4. ADMET and Drug-Likeness Analysis

To predict ADME using pkCSM, we used an online web server (http://biosig.lab.uq.edu.au/pkcsm/), accessed on 9 June 2023, while the T (toxicity) and Lipinski’s Rule of Five were predicted using the online web server Protox-II (https://tox-new.charite.de/protox_II/), accessed on 11 July 2023 [115]. Protox-II provides information regarding the prediction of the toxicity end point, using the canonical SMILES of the compound to assess toxicity [110].

## 5. Conclusions

The high rate of antibiotic resistance against *C. albicans* became an important basis for the discovery of new natural anti-fungal drugs. Based on the ethnopharmacological data and anti-fungal activity, guided isolation of Red Piper (*P. crocatum*) results in good anti-fungal constituents of new compounds **1** and **2**, which are reported here for first time, together with stigmasterol (**3**). According to the comprehensive analysis data of the in vitro assay and in silico study, compounds **1**–**3** showed strong binding activity and inhibition constants, are predicted to be safe as new drug candidates, and meet the five Ro5 parameters. Based on the finding research data, the promising novel anti-fungal constituents of *P. crocatum* can be proposed as new anti-fungal candidates to treat and cure fungal infections due to *C. albicans*.

## Figures and Tables

**Table 1:** NMR data of compound **1** (500 MHz for ^1^H and 125 MHz for ^13^C, in CDCl_3_).

No.	^13^C NMR	^1^H NMR
	δc	δ_H_ (Integral, Mult., J = Hz)
1	166.5	-
2	117.0	6.25 (1H, d, 16)
3	143.9	7.54 (1H, d, 16)
1′	127.4	-
2′	129.9	7.39 (1H, d, 8.5)
3′	115.9	6.83 (1H, d, 8.5)
4′	157.7	-
5′	115.9	6.83 (1H, d, 8.5)
6′	129.9	7.39 (1H, d, 8.5)
1″	83.1	-
2″	40.1	1.83 & 1.91 (1H, m)
3″	22.5	2.02 (2H, dd, 7.5; 16.25)
4″	123.9	5.10 (1H, t, 2)
5″	131.9	-
6″	25.8	1.66 (3H, s)
7″	17.7	1.60 (3H, s)
8″	23.8	1.60 (3H, s)
9″	141.9	6.02 (1H, dd, 11; 17.5)
10″	113.2	5.16 (2H, dd, 16.5; 11)
-	-	1.71
-	-	5.83

**Table 3:** NMR data of compound **3** (500 MHz for ^1^H and 125 MHz for ^13^C, in CDCl_3_).

No.	^13^C NMR	^1^H NMR
	δc	δ_H_ (Integral, Mult., J = Hz)
1	37.3	1.82 (2H, m)
2	31.9	1.5 9(2H, m)
3	71.9	3.51 (1H, m)
4	42.4	2.26 (2H, dd, 2:2)
5	140.8	-
6	121.8	5.33 (1H, t, 2)
7	31.7	1.98 (-)
8	31.9	1.59 (-)
9	50.2	0.99 (-)
10	36.2	-
11	21.3	1.44 (-)
12	39.7	1.28 (-)
13	42.3	-
14	56.9	1.03 (-)
15	24.4	1.50 (-)
16	29.0	1.23 (-)
17	56.0	1.14 (-)
18	12.0	0.65 (-)
19	19.1	0.99 (-)
20	40.62	1.23 (-)
21	23.1	1.05 (3H, d, 7)
22	138.4	5.13 (1H, dd, 8.5; 15)
23	129.3	5.00 (1H, dd, 8.5; 15.4)
24	51.3	1.50 (-)
25	31.9	1.49 (-)
26	21.2	0.78 (3H, d, 7.5)
27	19.5	0.83 (3H, d, 1)
28	25.5	1.15 (-)
29	12.1	0.83 (-)

**Table 4:** The antifungal activity of compounds **1**–**3** against *C. albicans* ATCC 10231.

Compounds	Inhibition Zone (mm) at Concentrations (% *w*/*v*)			Concentrations (% *w*/*v*)	
	2.5	5	10	MIC	MFC
Compound **1**	8.9	10.0	11.9	0.46	1.8
Compound **2**	9.4	12.4	13.0	0.62	2.5
Compound **3**	9.7	12.8	14.5	0.31	1.2
Ketoconazole (pc) [5,22]	30.0	31.3	32.2	0.00005	0.0001
Methanol (nc)	0	0	0	nm	nm
Sterile water (nc)	0	0	0	nm	nm

Note: nm = not measured; pc = positive control; nc = negative control.

**Table 5:** Prediction of antifungal activity of compounds **1**–**3** by binding affinity/ΔG (Kcal/mol) and inhibition constant/Ki (μM).

Ligands	Binding Affinity/ΔG (Kcal/mol)				Inhibition Constant/Ki (μM)			
	ERG1	ERG2	ERG11	ERG24	ERG1	ERG2	ERG11	ERG24
Positive Control	−7.36	−10.99	−10.09	−5.25	4.06	8.7 × 10^−3^	4 × 10^−2^	140.73
Ligand **1**	−7.86	−9.18	−8.38	−5.33	1.75	1.8 × 10^−1^	7.2 × 10^−1^	122.90
Ligand **2**	−6.77	−8.97	−7.68	−4.06	10.94	2.6 × 10^−1^	2.37	1060
Ligand **3**	−11.14	−12.78	−12.00	−6.89	6.8 × 10^−3^	4 × 10^−4^	1.6 × 10^-3^	8.88

Note: positive control for ERG 1: terbinafine, ERG11: ketoconazole, ERG2 and ERG24: amorolfine.

**Table 6:** Molecular interaction amino acid residues of ERG1, ERG2, ERG11, and ERG24.

Type of Interaction	Residues															
	ERG1				ERG2				ERG11				ERG24			
	Compounds				Compounds				Compounds				Compounds			
	Terbina- Fine	1	2	3	Amorol- Fine	1	2	3	Keto- Conazole	1	2	3	Amorol- Fine	1	2	3
Conventional	-	Asp B:408, Leu B:134	Ala B:284	Pro B:389	-	Glu B:172, Thr B:181	Asp B:126, Val B:152, Ile B:124	-	Tyr B:64	His B:377, Pro B:375, Met B:508	His B:377, Met B:508, Pro B:375	Gly B:307	**Tyr B:139**	**Tyr B:139**, Tyr B:45	Gln B:47, Tyr B:45	-
Carbon	Asp B:408	-	-	-	Asp B:126, Tyr B:120	-	-	-	-	Leu B:376	Ser B:507, Leu B:376	-	-	-	-	-
π–donor hydrogen bond					**Tyr B:103**	**Tyr B:103**	-	-	Ser B:378	-	-	-	-	-	-	-
π-sigma	Val B:163	-	-	-	**Tyr B:103**	His B:135, Phe B:107	His B:154, Tyr B:206	**Tyr B:103**, Phe B:107	Leu B:87	Phe B:380	-	-	**His B:288**	-	**His B:288**, Tyr B:45	Tyr B:46
π–π stacked	-	-	-	-	Ala B:98, Leu B:95, Tyr B:206, Ile B:178	Trp B:89, Trp B:164, Val B:162	-	-	-	His B:377	-	-	-	-	-	-
π–π T-shaped	-	-	-	-	-	-	-	-	-	-	-	-	-	Tyr B:45	-	-
Alkyl	Ala B:424, **Val B:133**	Ile B:162, Met B:388, Leu B:345, Phe B:306, Val B:163, Pro B:415	Leu B:287, Val B:163, Met B:388, Pro B:415, Ile B:162, Phe B:306, Leu B:345	**Val B:133**, Met B:421, Val B:163, Leu B:287, Pro B:145	**Trp B:164**, His B:154, **Trp B:89**, **Ile B:124**, **Val B:162**	**Val B:162**, Phe B:133	Ile B:178, Tyr B:120, Ala B:185, **Ile B:124**	**Trp B:164**, **Trp B:89**, Phe B:107	**Met B:508**, Val B:509, Thr B:311	Tyr B:401, Lys B:90	Phe B:228, Tyr B:118, Phe B:380, Phe B:233, Met B: 508, Leu B:376	His B:377	Tyr B:139, Leu B:223, His B:289	-	-	-
π-alkyl	Ile B:162, Met B:388, Pro B:415	Val B:133	Val B:133	-	**Ala B:185**	**Ala B:185**	Ile B:178, Leu B:105, Leu B:182, Tyr B:103, Thr B:181, Tyr B:120	Leu B:182, Tyr B:206, Leu B:95, Ala B:98, Tyr B:103	**Leu B:376**	Phe B:233, Val B:234, Leu B:88	-	**Leu B:376**	**His B:288**	Tyr B:46	Tyr B;45, Tyr B:46	Tyr B:46, Tyr B:45, **His B:288**
Van der Waals	**Phe B:306**, **Ala B:390**, **Pro B:389**, **Arg B:413**, **Gly B:420**, **Gly B:407**, **Met B:421**,	**Ala B:390**, Ser B:391, **Pro B:389**, **Arg B:413**, Met B:412, **Leu B:287**, Arg B:161, Gly B:286,	Gly B:135, **Gly B:420**, **Met B:421**, **Leu B:134**, Gly B:286, Gly B:132, Gly B:130,	Leu B:406, Ala B:409, **Gly B:407**, Gly B:286, **Leu B:134**, Ala B:284, Gly B:135,	**Glu B:172**, **Thr B:181**, **Leu B:182**, **Leu B:105**, **Ser B:117**	Thr B:160, Gln B:135, Val B:152, **Ser B:117**, Asp B:126, Val B:84, Tyr B:120,	Phe B:133, Val B:162, **Ser B:117**, Trp B:164, Phe B:107, Trp B:89, Leu B:95,	**Ser B:117**, Tyr B:120, Phe B:184, Val B:84, Ala B:185, **Thr B:181**, **Leu B:105**,	Gln B:66, **Gly B:65**, **Pro B:230**, His B:377, **Ser B:378**, **Phe B:228**, **His B:310**,	Met B:92, Leu B:87, Tyr B:64, **Ser B:507**, Val B:509, **Ser B:378**, **Pro B:230**,	Tyr B:64, Ser B:378, **Pro B:230**, **Phe B:126**, **Gly B:307**, Thr B:122, **Leu B:121**,	**Phe B:380**, **Pro B:230**, **Ser B:507**, **Ser B:378**, Pro B:375, Val B:509, **His B:310**,	Tyr B:45, **Thr B:44**, **Gln B:47**	**Thr B:44**, His B:65, Arg B:61, **Gln B:47**, His B:288	**Thr B:44**, Glu B:49, His B:65	His B:65, **Thr B:44**, Val B:287, Leu B:223, **Gln B:47**
	**Leu B:134**, **Leu B:287**, **Gly B:164**, Tyr B:335	Gly B:132, Gly B:135	Ala B:131, Asp B:285, Arg B:161, **Ala B:390**, **Pro B:389**, Asp B:408, **Gly B:407**, Ala B:424	Gly B:132, Arg B:161, Asp B:408, His B:226, Met B:388, **Phe B:306**, **Ala B:390**, Ser B:391, **Gly B:164**, **Gly B:420**, Ala B:424		**Leu B:182**, Ile B:178, **Leu B:105**, Ile B:124	Phe B:184, **Glu B:172**, Val B:153, Ser B:125	Met B:93, Ile B:178, Ala B:98, **Thr B:181**, **Glu B:172**, Asp B:126	**Phe B:126**, **Gly B:307**, **Thr B:311**, **Leu B:121**, Leu B:88, **Phe B:380**, **Phe B:233**, Lys B:90, **Ser B:507**, Tyr B:505, **Ser B:506**	**Lys B:90**, Ala B:117	Val B:509, Val B:510	**Thr B:311**, Gly B:308, **Phe B:228**, **Leu B:121**, Met B:508, **Phe B:233**, **Gly B:65**, Tyr B:64, **Tyr B:505**, **Ser B:506**, Leu B:87				

Note: text in **bold** showed the amino acid residues from compounds **1**–**3** that have similar molecular interactions with the positive control.

**Table 7:** ADMET prediction of ligands **1**, **2,** and **3**.

Pharmacokinetic Properties	Parameters	Ligands		
		1	2	3
Absorption	Water solubility	−4.674 log mol/L	−7.66 log mol/L	−6.673 log mol/L
	Intestinal absorption	89.583% absorbed	90.281% absorbed	96.151% absorbed
	Skin permeability	−2.7 log Kp	−2.792 log Kp	−2.781 log Kp
Distribution	Volume distribution (VDss)	0.366 log L/kg	0.11 log L/kg	0.18 log L/kg
	BBB permeability	−0.062 log BB	−0.414 log BB	0.799 log BB
	CNS permeability	−2.12 log PS	−1.36 log PS	−1.737 log PS
Metabolism	Inhibitor of:	Yes	Yes	No
	CYP1A2			
	CYP2C19	Yes	No	No
	CYP2C9	Yes	No	No
	CYP2D6	No	No	No
	CYP3A4	No	No	No
Excretion	Total Clearance	0.418 log mL/min/kg	1.861 log mL/min/kg	0.618 log mL/min/kg
Acute oral toxicity	Lethal dose 50%	2.331 mol/kg	1.654 mol/kg	2.375 mol/Kg
	Skin sensitization	No	Yes	No

**Table 8:** Physicochemical properties of ligands **1**, **2**, and **3** by drug-likeness Lipinski’s Rule of Five prediction.

Parameters	Ligands		
	1	2	3
Molecular mass (<500 Daltons)	299.41	325.57	412.69
Hydrogen bond donors/HBD (<5)	2	1	1
Hydrogen bond acceptors/HBA (<10)	3	2	1
LogP (<5)	4.6	7.35	7.8
Molecular refractivity (40–130)	93.52	106.38	132.76
Rotatable bonds	8	15	5
Topological polar surface area	49.33	35.25	20.23
Violation	0	1	2
Drug-likeness	Yes	Yes	No

## Data Availability

Data are contained within the article.
